# Screening for Lung Cancer — 10 States, 2017

**DOI:** 10.15585/mmwr.mm6908a1

**Published:** 2020-02-28

**Authors:** Thomas B. Richards, Ashwini Soman, Cheryll C. Thomas, Brenna VanFrank, S. Jane Henley, M. Shayne Gallaway, Lisa C. Richardson

**Affiliations:** ^1^Division of Cancer Prevention and Control, National Center for Chronic Disease Prevention and Health Promotion, CDC; ^2^Cyberdata Technologies, Atlanta, Georgia; ^3^Office on Smoking and Health, National Center for Chronic Disease Prevention and Health Promotion, CDC.

Lung cancer is the leading cause of cancer death in the United States; 148,869 lung cancer-associated deaths occurred in 2016 (*1*). Mortality might be reduced by identifying lung cancer at an early stage when treatment can be more effective (*2*). In 2013, the U.S. Preventive Services Task Force (USPSTF) recommended annual screening for lung cancer with low-dose computed tomography (CT) for adults aged 55–80 years who have a 30 pack-year* smoking history and currently smoke or have quit within the past 15 years (*2*).^†^ This was a Grade B recommendation, which required health insurance plans to cover lung cancer screening as a preventive service.^§^ To assess the prevalence of lung cancer screening by state, CDC used Behavioral Risk Factor Surveillance System (BRFSS) data^¶^ collected in 2017 by 10 states.** Overall, 12.7% adults aged 55–80 years met the USPSTF criteria for lung cancer screening. Among those meeting USPSTF criteria, 12.5% reported they had received a CT scan to check for lung cancer in the last 12 months. Efforts to educate health care providers and provide decision support tools might increase recommended lung cancer screening.

BRFSS is a random-digit–dialed landline and cellular telephone survey of the noninstitutionalized U.S. adult population aged ≥18 years conducted by state health departments in conjunction with CDC.^††^ In 2017, for the first time, an optional module added questions on lung cancer screening. In combination with core BRFSS questions on age and cigarette smoking status, three questions^§§^ from the lung cancer screening module enabled calculation of cigarette pack-years smoked. A fourth question asked about receipt of CT scans in the last 12 months with the following possible responses: “Yes, to check for lung cancer”; “No (did not have a CT scan)”; and “Had a CT scan, but for some other reason.”

Ten states administered this module to 85,514 respondents^¶¶^ during January 2017–December 2017.*** Weighted estimates were derived by following BRFSS recommendations for optional modules (*3*) and using SAS-callable SUDAAN (version 11.0; RTI International) to account for the BRFSS stratified, complex sampling design. Current, former, and never cigarette smoking status^†††^ and smoking pack-year categories^§§§^ were defined for adults aged 55–80 years^¶¶¶^ (33,137). Weighted prevalences of smoking status and pack-year categories among adults aged 55–80 years were estimated for each state. Weighted populations for smoking status and pack-year categories were derived by multiplying the state population of persons aged 55–80 years by the weighted percentage of adults in the corresponding smoking status and pack-year category.

Weighted estimates were calculated for self-reported receipt of lung cancer screening among current and former cigarette smokers (hereafter referred to as smokers) aged 55–80 years (14,585) who did and did not meet USPSTF criteria for lung cancer screening.**** The weighted prevalence of lung cancer screening was calculated by age group, smoking status, sex, race/ethnicity, education, general health status, health care coverage, routine checkup in the past year, and diagnosed chronic obstructive pulmonary disease (COPD), emphysema, or bronchitis.^††††^ Logistic regression was used to calculate prevalence ratios (PRs) with 95% confidence intervals, with reported lung cancer screening as the outcome, adjusted for all other variables.

Overall, 12.7% of adults aged 55–80 years met USPSTF criteria for lung cancer screening, with nearly one half (5.6%) being former smokers (Table 1). The percentage of adults meeting USPSTF screening criteria by state ranged from 8.9% (Maryland) to 17.0% (Oklahoma). The population size meeting USPSTF criteria ranged from 16,200 (Vermont) to 610,000 (Florida) (Supplementary Table 1, https://stacks.cdc.gov/view/cdc/85167).

**TABLE 1:** Estimated prevalence of adults* aged 55–80 years who met^†^ and did not meet^§^ U.S. Preventive Services Task Force (USPSTF) lung cancer screening criteria, by pack-years (PYs)^¶^ — 10 states, Behavioral Risk Factor Surveillance System, 2017

State	Total no.** of adults aged 55–80 yrs	%^††^ (95% CI)									
		Met USPSTF criteria			Did not meet USPSTF criteria						Never smokers
		Current ≥30 PY smokers	Former ≥30 PY smokers, quit <15yrs	Total who met criteria	Current 20–29 PY smokers	Current <20 PY smokers	Former ≥30 PY smokers, quit ≥15yrs	Former 20–29 PY smokers	Former <20 PY smokers	Total who did not meet criteria	
Florida	7,936	7.2 (6.1–8.4)	5.8 (4.8–7.0)	13.0 (11.5–14.5)	3.4 (2.6–4.6)	3.7 (2.9–4.8)	3.7 (3.1–4.6)	4.4 (3.7–5.4)	17.4 (15.6–19.5)	32.8 (32.5–35.2)	54.2 (51.8–56.7)
Georgia	2,123	6.0 (4.9–7.5)	3.7 (2.9–4.9)	9.8 (8.3–11.5)	3.0 (2.2–4.1)	5.3 (4.2–6.6)	3.9 (2.9–5.0)	4.2 (3.2–5.4)	15.7 (13.8–17.7)	31.9 (29.5–34.5)	58.3 (55.6–60.9)
Kansas	4,175	7.1 (6.2–8.2)	6.4 (5.5–7.4)	13.5 (12.3–14.8)	3.3 (2.7–4.0)	3.7 (3.0–4.6)	4.7 (4.0–5.6)	4.0 (3.4–4.8)	14.6 (13.4–15.9)	30.4 (28.8–32.1)	56.1 (54.3–57.9)
Maine	1,680	7.3 (5.5–9.5)	5.8 (4.3–7.8)	13.1 (10.8–15.8)	4.2 (3.0–6.0)	3.3 (2.2–5.0)	5.9 (4.6–7.6)	5.7 (4.4–7.5)	21.5 (19.0–24.4)	40.7 (37.4–44.0)	46.3 (42.9–49.7)
Maryland	5,724	4.7 (3.9–5.6)	4.2 (3.6–5.0)	8.9 (7.9–10.0)	2.3 (1.8–2.9)	3.2 (2.7–4.0)	3.7 (3.1–4.4)	4.7 (4.1–5.5)	17.5 (16.2–18.9)	31.4 (29.8–33.2)	59.7 (57.8–61.5)
Missouri	2,896	9.2 (7.7–11.1)	7.4 (6.2–8.8)	16.7 (14.7–18.8)	2.7 (2.0–3.6)	3.7 (2.8–4.8)	3.6 (2.9–4.5)	4.7 (3.8–5.9)	13.4 (11.8–15.2)	28.1 (25.9–30.4)	55.3 (52.8–57.8)
Nevada	1,431	8.5 (6.2–11.7)	4.5 (3.2–6.2)	13.0 (10.3–16.3)	3.4 (2.0–5.7)	7.6 (5.5–10.4)	3.9 (2.8–5.5)	4.5 (3.1–6.5)	14.9 (12.2–18.0)	34.3 (30.4–38.4)	52.7 (48.5–56.9)
Oklahoma	2,520	8.6 (7.3–10.1)	8.4 (7.1–9.9)	17.0 (15.2–19.0)	3.2 (2.4–4.2)	2.9 (2.2–3.8)	4.8 (3.9–5.9)	4.1 (3.3–5.1)	13.7 (12.2–15.4)	28.7 (26.6–30.9)	54.3 (51.9–56.6)
Vermont	2,667	5.0 (4.0–6.2)	5.7 (4.7–7.1)	10.8 (9.3–12.4)	3.0 (2.3–3.9)	2.9 (2.2–3.9)	3.6 (2.8–4.5)	4.7 (3.8–5.7)	22.9 (20.9–25.0)	37.0 (34.7–39.4)	52.2 (49.8–54.6)
Wyoming	1,985	7.4 (6.1–9.0)	5.4 (4.4–6.8)	12.8 (11.1–14.7)	3.5 (2.6–4.7)	3.7 (2.9–4.9)	5.7 (4.6–7.2)	4.9 (3.9–6.2)	15.8 (14.0–17.7)	33.7 (31.3–36.2)	53.5 (50.9–56.1)
**Total**	**33,137**	**7.1 (6.5–7.7)**	**5.6 (5.1–6.2)**	**12.7 (12.0–13.4)**	**3.2 (2.7–3.7)**	**4.0 (3.6–4.5)**	**4.0 (3.6–4.4)**	**4.5 (4.1–4.9)**	**16.4 (15.5–17.3)**	**32.1 (86.5–88.0)**	**55.3 (54.1–56.5)**

**Abbreviations:** CI = confidence interval; smokers = cigarette smokers.

*Respondents were excluded if missing age, smoking status, PY information, or if there was a history of prior cancer diagnosis.

^†^ Met USPSTF lung cancer screening criteria: adults aged 55–80 years who had a ≥30 PY cigarette smoking history and currently smoke or quit <15 years ago.

^§^ Did not meet USPSTF lung cancer screening criteria: adults aged 55–80 years who smoked cigarettes but had <30 PY smoking history or quit ≥15 years ago, or who were never smokers.

^¶^ The average of number of 20-cigarette packs smoked per day multiplied by the number of years smoked.

** Unweighted number of respondents.

^††^ Weighted percentage of row total.

Lung cancer screening was reported by 12.5% of smokers who met USPSTF criteria and ranged from 9.7% (Oklahoma) to 16.0% (Florida) (Table 2). Differences between states ranged from –3.8% (Oklahoma versus Vermont) to 6.3% (Florida versus Oklahoma) (Supplementary Table 2, https://stacks.cdc.gov/view/cdc/85168). Lung cancer screening was reported by 7.9% of smokers aged 55–80 years who did not meet USPSTF criteria and ranged from 4.3% (Maryland) to 9.4% (Oklahoma and Florida) (Table 2). Differences between states ranged from –5.1% (Maryland versus Oklahoma) to 5.2% (Florida versus Maryland) (Supplementary Table 2, https://stacks.cdc.gov/view/cdc/85168).

**TABLE 2:** Prevalence of receipt of a computed tomography (CT) scan to check for lung cancer, reported among smokers* aged 55–80 years who met^†^ and did not meet^§^ U.S. Preventive Services Task Force (USPSTF) lung cancer screening criteria — 10 states, Behavioral Risk Factor Surveillance System, 2017

State	Met USPSTF criteria			Did not meet USPSTF criteria		
	Total no.^¶^	Received CT scan to check for lung cancer		Total no.^¶^	Received CT scan to check for lung cancer	
		No.**	%^††^ (95% CI)		No.**	%^††^ (95% CI)
Florida	1,115	147	16.0 (11.8–21.3)	2,525	219	9.4 (7.3–12.2)
Georgia	187	21	—^§§^	650	54	7.6 (5.4–10.6)
Kansas	506	56	10.5 (8.0–13.8)	1,208	81	6.9 (5.3–8.8)
Maine	194	21	—^§§^	659	36	5.1 (3.2–8.0)
Maryland	517	49	11.2 (7.4–16.5)	1,861	73	4.3 (3.2–5.7)
Missouri	421	55	10.5 (7.4–14.7)	867	74	8.5 (6.2–11.4)
Nevada	179	14	—^§§^	503	23	—^§§^
Oklahoma	364	39	9.7 (6.7–13.7)	714	61	9.4 (7.0–12.4)
Vermont	265	40	13.5 (9.1–19.6)	980	56	5.9 (4.2–8.2)
Wyoming	227	22	—^§§^	643	27	—^§§^
**Total**	**3,975**	**464**	**12.5 (10.4–14.9)**	**10,610**	**704**	**7.9 (6.8–9.1)**

**Abbreviation:** CI = confidence interval.

* Includes both current and former cigarette smokers aged 55–80 years. Respondents were excluded if never smokers, if missing age, smoking status, or pack-year (average number of 20-cigarette packs smoked per day multiplied by number of years smoked) information, or if they had a history of a prior cancer diagnosis.

^†^ Met USPSTF lung cancer screening criteria: adults aged 55–80 years who had a ≥30 pack-year smoking history and who currently smoke or quit <15 years ago.

^§^ Did not meet USPSTF lung cancer screening criteria: adults aged 55–80 years who smoked but had <30 pack-year smoking history or quit ≥15 years ago.

^¶^ Unweighted number of smokers.

** Unweighted number of smokers who reported a CT scan to check for lung cancer.

^††^ Weighted percentage.

^§§^ Proportions were suppressed where relative standard error ≥30% or n<30.

Among smokers who met USPSTF criteria, higher prevalence of lung cancer screening was associated with diagnosed COPD (PR = 3.01) and lower prevalence of screening with no routine checkup in the past year (PR = 0.54) (Table 3). Among smokers who did not meet USPSTF criteria, higher prevalence of screening was associated with diagnosed COPD (PR = 2.34) and lower prevalences of screening with being a former smoker (PR = 0.63) and fair or poor general health status (PR = 0.68).

**TABLE 3:** Characteristics associated with reported receipt of a computed tomography (CT) scan to check for lung cancer among smokers* aged 55–80 years who met^†^ and did not meet^§^ U.S. Preventive Services Task Force (USPSTF) lung cancer screening criteria — 10 states, Behavioral Risk Factor Surveillance System, 2017

Characteristic	Smokers who met USPSTF lung cancer screening criteria				Smokers who did not meet USPSTF lung cancer screening criteria			
	Total no.^¶^	No.**	%^††^ (95% CI^§§^)	PR^¶¶^ (95% CI^§§^)	Total no.^¶^	No.**	%^††^ (95% CI^§§^)	PR^¶¶^ (95% CI^§§^)
**Age group (yrs)**								
55–64	2,052	211	11.3 (8.9–14.4)	Referent	4,299	261	7.2 (5.7–9.0)	Referent
65–80	1,851	253	14.8 (11.3–19.1)	0.95 (0.68–1.33)	6,143	443	8.8 (7.2–10.6)	1.26 (0.89–1.80)
**Smoking status**								
Current smokers	2,120	254	12.6 (9.8–16.1)	Referent	2,062	194	11.1 (8.0–15.1)	Referent
Former smokers	1,783	210	13.1 (10.0–16.9)	0.99 (0.72–1.37)	8,380	510	7.1 (6.1–8.3)	0.63 (0.41–0.95)
**Sex**								
Male	2,140	256	12.4 (9.6–15.8)	Referent	4,907	325	8.7 (6.9–10.9)	Referent
Female	1,761	208	13.5 (10.5–17.3)	0.90 (0.64–1.28)	5,531	379	7.2 (6.1–8.6)	0.75 (0.56–1.00)
**Race/Ethnicity**								
White, non-Hispanic	3,425	400	12.9 (10.7–15.5)	Referent	8,704	571	8.1 (6.9–9.4)	Referent)
Other	414	51	—***	—***	1,570	126	8.2 (5.6–11.9)	0.91 (0.63–1.37)
**Education**								
High school or less	1,938	225	12.5 (9.5–16.2)	Referent	3,731	278	8.9 (7.1–10.9)	Referent
College or technical school	1,958	238	13.3 (10.4–16.7)	1.19 (0.85–1.69)	6,693	426	7.5 (6.1–9.1)	1.05 (0.76–1.46)
**General health status**								
Excellent/Very good/Good	2,433	222	10.9 (8.3–14.3)	Referent	8,133	438	6.6 (5.5–7.9)	Referent
Fair/Poor	1,462	240	16.2 (12.9–20.1)	0.86 (0.59–1.27)	2,282	265	12.3 (9.5–15.8)	0.68 (0.48–0.95)
**Have health care coverage**								
Yes	3,577	448	13.7 (11.4–16.4)	Referent	9,913	672	8.2 (7.0–9.5)	Referent
No	313	13	—***	—***	510	31	5.7 (2.7–11.7)	0.55 (0.30–1.02)
**Routine check-up in the past year**								
Yes	3,036	411	14.4 (11.9–17.4)	Referent	8,630	622	8.3 (7.1–9.7)	Referent
No	783	44	5.6 (3.4–9.7)	0.54 (0.32–0.90)	1,672	69	5.6 (3.4–9.0)	0.78 (0.46–1.32)
**Ever told have chronic obstructive pulmonary disease, emphysema, or chronic bronchitis**								
Yes	1,410	283	23.4 (19.1–28.4)	3.01 (2.02–4.49)	1,496	224	17.3 (13.8–21.5)	2.34 (1.70–3.22)
No	2,467	180	6.8 (5.2–8.9)	Referent	8,876	472	6.5 (5.4–7.8)	Referent

**Abbreviations:** CI = confidence interval; PR = prevalence ratio.

* Includes both current and former cigarette smokers. Respondents were excluded if never smokers, if missing age, smoking status, or pack-year (average number of 20-cigarette packs smoked per day multiplied by the number of years smoked) information, or if history of prior cancer diagnosis.

^†^ Met USPSTF lung cancer screening criteria: adults aged 55–80 years who had a ≥30 pack-year smoking history and currently smoke or quit <15 years ago.

^§^ Did not meet lung cancer screening USPSTF criteria: adults aged 55–80 years who smoked but had <30 pack-year smoking history or quit ≥15 years ago.

^¶^ Unweighted total number of current and former cigarette smokers aged 55–80 years with the row characteristic.

** Unweighted number of current and former cigarette smokers aged 55–80 years who reported lung cancer screening within this USPSTF category.

^††^ Weighted percentage.

^§§^ 95% CIs generated using logit transformation in SUDAAN.

^¶¶^ Model-adjusted, with adults aged 55–80 years who smoked and reported lung cancer screening as the outcome and adjusted for all other variables listed in the table.

*** Proportions are suppressed where relative standard error ≥30% or n<30.

## Discussion

These findings suggest an opportunity to educate both patients and health care providers, provide decision support tools to reinforce appropriate screening triage, and apply evidence-based interventions from The Community Guide.^§§§§^ Previous studies have used data from the 2017 BRFSS optional module on lung cancer screening to analyze utilization by state (*4*) and among sexual minorities (*5*). This report adds information about the prevalence and population size by different categories of pack-year smoking for each participating state. The 2017 BRFSS questions to identify smoking pack-years were similar to those that a health care provider might ask in a clinic.

States can use the BRFSS lung cancer screening estimates to identify where increased screening is needed, to develop supplementary research projects to evaluate barriers to screening, ^¶¶¶¶^ and to monitor the effectiveness of interventions.***** Annual lung cancer screening is a secondary preventive health care strategy (*2*). The most effective primary preventive measures for lung cancer are to never start smoking and for smokers to stop cigarette smoking as soon as possible (*6*). The National Cancer Institute and the Veterans Health Administration are currently supporting clinical trials to test smoking cessation intervention strategies for patients undergoing lung cancer screening (*7*). Evidence-based tobacco cessation interventions in the 2008 U.S. Public Health Service clinical guidelines and The Community Guide include advising patients to quit smoking, providing cessation counseling and medications, and connecting patients to other cessation resources such as 1–800-QUIT-NOW (*6*). Recent studies suggest that training primary care providers on how to conduct shared decision-making discussions and implement effective smoking cessation interventions in the context of lung cancer screening is needed (*7*,*8*).

Cigarette smoking is the leading cause of COPD in the United States (*9*). In the current report, diagnosed COPD was associated with a higher prevalence of lung cancer screening, both in smokers who met and those who did not meet USPSTF criteria. The severity of the reported COPD is unknown. The USPSTF does not recommend lung cancer screening if a health problem is present that would substantially limit life expectancy or the ability to undergo curative lung surgery (*2*).

The findings in this report are subject to at least four limitations. First, lung cancer screening and smoking were self-reported without medical record validation. Self-reported smoking history might be subject to recall bias and social desirability bias. Some respondents might not know the exact type of test they received or the reason that their doctor ordered the test. Second, the 2017 BRFSS module does not address whether current smokers were provided with cessation counseling and medications. Third, the module does not provide information on health care resources that might differ by location within a state, such as the distribution of American College of Radiology–accredited lung cancer screening facilities.^†††††^ Finally, some caution might be needed when comparing these results with those from other surveys. For example, the prevalence of screening in the 2017 BRFSS among adults who met USPSTF criteria (12.5%) was higher than that reported in the 2015 National Health Interview Survey (4.4%) (*10*). Although an actual increase in screening delivery likely occurred from 2015 to 2017, differences in methods of data collection, question wording, and populations covered could result in different estimates.

Public health initiatives to prevent cigarette smoking, increase smoking cessation, and increase lung cancer screening among those who meet USPSTF criteria could help reduce lung cancer mortality. Avoidance of screening inconsistent with USPSTF criteria could reduce the potential for harms such as overdiagnosis and overtreatment (*10*). Efforts to educate^§§§§§^ health care providers regarding the benefits of lung cancer screening and to provide decision support tools^¶¶¶¶¶^ might increase appropriate and timely lung cancer screening.

**Summary**

**What is already known about this topic?**

The U.S. Preventive Services Task Force (USPSTF) recommends annual lung cancer screening for adults aged 55–80 years who have a ≥30 pack-year cigarette smoking history and currently smoke or have quit <15 years ago.

**What is added by this report?**

In 10 states, one in eight persons aged 55–80 years met USPSTF criteria, and, among those meeting USPSTF criteria, only one in eight reported a lung cancer screening exam in the last 12 months.

**What are the implications for public health practice?**

Public health initiatives to prevent cigarette smoking, increase smoking cessation, and increase recommended lung cancer screening could help reduce lung cancer mortality.

## Footnotes

* Pack-years (PYs) are the average of number of 20-cigarette packs smoked per day multiplied by the number of years smoked.

† USPSTF is currently conducting a review and update of this recommendation.

§ Under Section 2713 of the Patient Protection and Affordable Care Act (ACA), individual and group health insurance plans must provide coverage of evidence-based screening services that have a rating of A or B in the current USPSTF recommendations and may not impose cost-sharing (such as copayments, deductibles, or co-insurance) on patients receiving these services. In addition, starting in 2015, the Centers for Medicare and Medicaid Services covered lung cancer screening for Medicare beneficiaries aged 55–77 years who met the USPSTF smoking criteria.

¶ https://www.cdc.gov/brfss/questionnaires/pdf-ques/2017_BRFSS_Pub_Ques_508_tagged.pdf.

** Florida, Georgia, Kansas, Maine, Maryland, Missouri, Nevada, Oklahoma, Vermont, and Wyoming.

†† https://www.cdc.gov/brfss/about/index.htm.

§§ ^“^You’ve told us that you have smoked in the past or are currently smoking. The next questions are about screening for lung cancer. Q1. How old were you when you first started to smoke cigarettes regularly? (enter: age in years). Q2. How old were you when you last smoked cigarettes regularly? (enter: age in years). Q3. On average, when you {smoke/smoked} regularly, about how many cigarettes {do/did} you usually smoke each day? (enter: number of cigarettes).”

¶¶ 2017 BRFSS combined landline and cellular telephone cooperation and response rates. https://www.cdc.gov/brfss/annual_data/2017/pdf/2017-response-rates-table-508.pdf.

*** All 10 states asked all respondents (including never smokers) Question (Q) 1 to Q3 on pack-years. Six states (Florida, Georgia, Maine, Maryland, Vermont, and Wyoming) asked all respondents (including never smokers) Q4 on lung cancer screening, and four states (Kansas, Missouri, Nevada, and Oklahoma) only asked current or former smokers (excluding never smokers) to respond to Q4.

††† Respondents were categorized as never smokers if they had smoked fewer than 100 cigarettes in their lifetime. Former smokers had smoked at least 100 cigarettes but now smoked cigarettes “not at all.” Current smokers had smoked at least 100 cigarettes and now smoked cigarettes “every day” or “some days.”

§§§ For current and former cigarette smokers who met USPSTF criteria for lung cancer screening, PY categories were 1) current smokers ≥30 PY and 2) former smokers ≥30 PY who had quit smoking <15 years ago. For current and former smokers who did not meet USPSTF criteria, the smoking PY categories were 1) current smokers 20–29 PY; 2) current smokers <20 PY; 3) former smokers >30 PY who quit smoking ≥15 years ago; 4) former smokers 20–29 PY; and 5) former smokers <20 PY.

¶¶¶ A restricted file with single-year age was used to determine years that smokers quit smoking for former smokers and to determine respondents aged 80 years. The public-use BRFSS combines persons aged ≥81 years with those aged 80 years into a single category aged ≥80 years.

**** The following categories and number of respondents were excluded: history of any cancer or missing smoking PY data (18,267); never smokers (41,537); age <55 years (9,661); and age >80 years (1,464).

†††† Diagnosed COPD was determined by a “yes” response to the question “Has a doctor, nurse, or other health professional ever told you that you had chronic obstructive pulmonary disease or COPD, emphysema, or chronic bronchitis?”

§§§§ The Community Guide (The Guide to Community Preventive Services; https://www.thecommunityguide.org/) does not directly address lung cancer screening but describes evidence-based interventions that could be applied. Examples include prevention and control of tobacco use; promoting informed decision making; provider reminder and recall systems; reducing structural barriers; small media targeting clients; one-on-one education for clients; and client out-of-pocket costs.

¶¶¶¶ The Maine Comprehensive Cancer Control Program has used surveys of lung cancer screening facilities to identify barriers to screening such as limited staffing, lack of patient and provider education, screening costs, and data reporting requirements. https://nccd.cdc.gov/nccdsuccessstories/showdoc.aspx?s=14224&dt=0.

***** https://www.cdc.gov/cancer/ncccp/pdf/LungCancerScreeningPrograms.pdf.

††††† https://www.acraccreditation.org/accredited-facility-search.

§§§§§ https://www.lucatraining.org/course.

¶¶¶¶¶ https://effectivehealthcare.ahrq.gov/decision-aids/lung-cancer-screening/clinicians-checklist.html.
